# Ataxia Rating Scales Reveal Increased Scores in Very Preterm Born 5–6-Year-Old Preschool Children and Young Adults

**DOI:** 10.1007/s12311-022-01463-8

**Published:** 2022-08-26

**Authors:** Bilge Albayrak, Anne-Kathrin Dathe, Katharina Maria Heuser-Spura, Ursula Felderhoff-Mueser, Dagmar Timmann, Britta Maria Huening

**Affiliations:** 1 Department of Pediatrics I, Neonatology, Pediatric Intensive Care and Pediatric Neurology, University Hospital Essen, University of Duisburg-Essen, Essen, Germany; 2 Center for Translational Neuro- and Behavioral Sciences, University Hospital Essen, University of Duisburg-Essen, Hufelandstraße 55, 45147 Essen, Germany; 3 Department of Neurology, University Hospital Essen, University of Duisburg-Essen, Essen, Germany

**Keywords:** Very preterm children, Very preterm adults, Ataxia rating scales, Difficulties in coordination and balance

## Abstract

The aim of this study is to investigate whether scores in ataxia rating scales (ARS) are different in very preterm (VP) preschool and adult participants compared to term controls. This is a case–control study. Sixty VP children (years: 5.5–6.5; gestational age: 23.9–31.7 weeks) and 56 VP adults (years: 17.8–27.9; gestational age: 23.3–32.0 weeks) without major cerebral lesions participated in the study; 60-age and sex-matched term children and 64 term adults for comparison were used in the study intervened with the assessment with International Cooperative Ataxia Rating Scale (ICARS) and Scale for Assessment and Rating of Ataxia (SARA). Main outcome measures are primary outcome: total icars and sara scores in preterm (vp) participants versus controls. Results showed that VP children showed significantly higher total ICARS (*M* 15.98, SD 6.29, range 4.0–32.0; *p* < .001) and SARA scores (*M* 6.5, SD 2.53, range 1.0–15.0; *p* < .001) than controls (ICARS: *M* 9.17, SD 3.88, range 2.0–20.0; SARA: *M* 3.51, SD 1.54, range 1.0–8.0). VP adults also showed significantly higher total ICARS (*M* 1.0, *SD* 1.99, range 0.0–11.0; p < .001) and SARA scores (*M* 0.54, SD 1.08, range 0.0–6.0; *p* < .001) than controls (ICARS: *M* 0.11, SD 0.44, range 0.0–2.0; SARA: *M* 0.04, *SD* 0.18, range 0.0–1.0). In conclusion, VP children showed significantly higher scores in ARS than controls. These differences were also present in VP adults, suggesting that deficits likely prevail until adulthood. ARS are a time and cost-effective method to screen for difficulties in coordination and balance in a patient group at risk.

## Introduction

Worldwide 5–18% of newborns are born preterm, of whom 1.5% are born with a very low birth weight (VLBW) (birthweight $\le$ 1500 g) [1]. The survival rate has significantly increased over the past decades due to progress in obstetric and neonatal care [2]. Furthermore, the incidence of structural brain injury with major motor deficits such as cerebral palsy (CP) has decreased [3]. Diffuse white matter injury with involvement of cortex, grey matter, and cerebellum to various degrees is currently the predominant type of brain injury also leading to aberrant brain development, known as “encephalopathy of prematurity” [3]. Very preterm birth ($\le$ 32 weeks of gestation, (wks)) is associated with high risk of neurodevelopmental deficits regarding cognition, social-emotional behavior, gross, fine, and visual-motor skills [3–8].

The cerebellum is responsible for motor control, in particular, balance and limb coordination, and motor learning [9, 10]. The fetal cerebellum shows the strongest and expeditious growth and differentiation during the second and third trimester of pregnancy [9]. Preterm birth occurs during this vulnerable phase, increasing the risk of structural tissue damage through hemorrhage or ischemia. In addition, and more importantly, there is a high risk of aberrant cerebellar development [10].

There is recent evidence that cerebellar-dependent motor learning is impaired in preterm children. For instance, very preterm (VP) children and adults showed reduced acquisition of conditioned eyeblink responses [11].

To evaluate cerebellar motor impairment in a clinical setting, ataxia rating scales (ARS) are used, developed for standardized quantification and disease monitoring of cerebellar ataxia of various etiologies [12–14]. The two most commonly used scales are the International Cooperative Ataxia Rating Scale (ICARS) and the Scale for Assessment and Rating of Ataxia (SARA) [13–15]. It has recently been reported that age-appropriate developing children show physiologically higher scores than adults in ARS up to an age of 12 years [16, 17]. To date, these scales have not been applied to VP children.

In VP children without CP, the “red flags” of neurological impairments such as abnormal muscle tone and strength and pathological reflexes are scarce; however, they often have difficulties in coordination, balance, fine and gross motor skills, with soft neurological signs such as clumsiness, mirror movements, reduced speed, and fluency [3, 18–20]. These soft signs are referred to as minor neurological disorder (MND) or developmental coordination disorder (DCD). Both disorders are not clearly defined but have a variable and multifactorial pathophysiology. They describe minor motor problems, but strong enough to affect everyday activities and participation [18, 21, 22]. Soft neurological signs may be easily overlooked in a clinical setting. Specific instruments like the Movement Assessment Battery for Children, Second Edition (M-ABC-2) and Bruininks-Oseretsky Test of Motor Proficiency, Second Edition (BOT-2) are time-consuming and resource intensive [23]. ARS may be a helpful additional instrument to screen for these soft signs as they are standardized, quantifiable, and easy to apply.

The aim of the present study was to provide first proof-of-principle data that preterm birth results in abnormal ARS scores in preschool-age children and adults. If this would be the case, application of ARS would be a helpful tool to identify those patients in need of further and more comprehensive testing. Since one scale (ICARS) is more detailed than the other (SARA), the aim was to investigate whether both could equally detect group differences in coordination and balance between preterm participants and controls.

## Methods

### Participants

As part of three previous studies conducted at the University Hospital Essen, Germany [8, 11], VP preschool-age children (born 2010–2012) and young adults (born 1990–2003) were recruited and compared to age- and sex-matched controls.

The inclusion criteria were: VP birth ($\le$32 wks) or term birth (($\ge$37 wks), and age-appropriate development of the participants without special needs (tested with WISC- Wechsler Preschool and Primary Scale of Intelligence-Third Edition and WAIS-Wechsler Adults Intelligence Scale- Fourth Edition). Exclusion criteria were intra-/(peri) ventricular hemorrhage ≥ III, periventricular leukomalacia, and neurological and skeletal disorders that could interfere with mental performance and motor coordination [8, 11].

For detailed clinical description of the included participants, see Tables 1 and 2.

**Table 1:** Group characteristics of very preterm children and controls

	Very preterm (*n* = 60)	Term (*n *= 60)	*p*^a^
Clinical characteristics			
Gestational age, weeks [range]	28.7 [23.9–31.7]	39.5 [38.0–42.0]	** < .001**
Birth weight, gram [range]	1126.0 [430–1860]	3414.3 [2380–4895]	** < .001**
SGA, *n* (%)	8 (13%)	5 (8%)	.378
Female, *n*	30	30	1.0
IVH < grade III, *n* (%)	4	0	.119
BPD, *n* (%)	7	0	**.013**
ROP < grade III, *n* (%)	13	0	** < .001**
Surgery, *n* (%)	15	1	** < .001**
Proven sepsis, *n* (%)	12	1	**.001**
Follow-up characteristics			
Age at assessment, years [range]	5.9 [5.5–6.5]	5.9 [5.5–6.5]	.681
Parental education, high^b^, *n* (%)	52^c^ (90%)	57^d^ (95%)	.813
IQ	96.6 [65.0–135.0]	102.1 [61.0–139.0]	**.041**
M-ABC 2 fine motor skills^g^	7.1 [1.7–12.8]	9.4 [4.3–12.7]	** < .001**
Any therapies^h^, *n* (%)	53 (88.3%)	23 (38.3%)	** < .001**
Total abnormality score (TAS)	2.9^e^ [1.0–11.0]		
Transcerebellar diameter (TCD)	50.9^f^ [42.2–56.3]		

Data are presented as mean (standard deviation) if not indicated otherwise

^a^*t* test and chi-square results for continuous and categorical data, respectively, statistically significant values are given in bold.

^b^ > 10 years school

^c^*n* = 58. ^d^*n* = 59. ^e^*n* = 31. ^f^*n* = 30

^g^Standard score of the Movement Assessment Battery for Children—second edition, subtask manual dexterity

^h^Having any therapies, including speech therapy, physical therapy, occupational therapy, or early support therapy

*SGA* small for gestational age (birth weight < 10th percentile), *IVH* intraventricular haemorrhage, *BPD* bronchopulmonary dysplasia, *ROP* retinopathy of prematurity, *IQ* intelligence quotient based on Snijders-Oomen Nonverbal Intelligence Test SON-R 2½–7, reasoning subscale, M-ABC 2—Movement Assessment Battery for Children—second edition, standard score

TAS—TAS was applied according to Dewan ert al. (minimum score = 0; maximum score = 21)[34]

TCD—TCD was applied according to Kidokoro et al.; TCD ≥ 50 mm was classified as normal, cutoff values for mild growth impairment were < 50 mm but ≥ 47 and < 47 mm for severe growth impairment [35]

**Table 2:** Group characteristics of very preterm adults and controls

	Very preterm (*n*= 56)	Term (*n* = 64)	*p*^a^
Clinical characteristics			
Gestational age, weeks [range]	29.2 [23.3–32.0]	39.9 [37.0–42.0]	** < .001**
Birth weight, gram [range]	1247 [520–2370]	3598^c^ [2540–5360]	** < .001**
SGA, *n*	7 (12.5%)	3^d^ (4.7%)	.187
Female, *n* (%)	25 (44.6%)	35 (54.7%)	.272
IVH < grad III, *n* (%)	8^e^ (14.3%)	0^f^ (0%)	**.002**
BPD, *n* (%)	16^e^ (28.6%)	0^ g^ (0%)	** < .001**
ROP, *n* (%)	14^ h^ (25.0%)	0^ g^ (0%)	** < .001**
Surgery, *n* (%)	12^i^ (21.4%)	2^f^ (81.3%)	**.002**
Proven sepsis, *n* (%)	20^ h^ (35.7%)	1^j^ (1.6%)	** < .001**
Follow-up characteristics			
Age at assessment, years [range]	20.1 [17.8–27.9]	21.6 [17.7–29.3]	**.001**
Education, high^b^, *n*	40	52^d^	.149
Parental education, high^b^, *n*	33	41^e^	.292
Health status^m^	5.5^ h^ [3.7–6.6]	5.8^ h^ [3.6–7.0]	.067
Cerebellum volume, (ml)	141.3^i^ [109.0–169.8]	144.5^ k^ [99.7–172.6]	.307
IQ	99.6^ h^ [80.0–123.0]	106.9^ h^ [81.0–123.0]	** < .001**
Any therapy^n^, *n *(%)	19^ l^ (40.4%)	3^ h^ (5.9%)	** < .001**
Any psychiatric disorders^o^, *n* (%)	13^ l^ (27.7%)	3^ h^ (5.9%)	**.004**

Data are presented as mean (standard deviation) if not indicated otherwise

^a^*t* test or Mann–Whitney *U* test and chi-square results or Fischer’s exact test for continuous and categorical data, respectively, statistically significant values are given in bold, ^b^ > 10 years school, ^c^*n* = 62**,** ^d^*n* = 63**,** ^e^*n* = 52, ^f^*n* = 58, ^g^*n* = 61, ^h^*n* = 51, ^i^*n* = 50, ^j^*n* = 59, ^k^*n* = 44, ^l^*n* = 47, ^m^assessment of health status based on the Life Satisfaction Questionnaire (Fragebogen zur Lebenszufreidenheit, FLZ), ^n^having any therapies, including speech therapy, physical therapy, or occupational therapy**,** ^o^psychiatric disorders including attention-deficit-(hyperactivity)-disorder, emotional disorder

*SGA* **s**mall for gestational age (birth weight < 10th percentile), *IVH* intraventricular haemorrhage, *BPD* bronchopulmonary dysplasia, *ROP* retinopathy of prematurity, *IQ* Intelligence quotient based on the Wechsler Adult Intelligence Scale–third edition

### Standard Protocol Approvals and Patient Consents

All participants and parents of children gave written informed consent. The Ethics committees of the University of Duisburg-Essen and the Heinrich-Heine University of Duesseldorf approved the studies (16–7265-BO, 2,017,074,357, 15–6181-BO, 19–8890-BO).

### Outcome Measures

Two ARS were used to identify deficits in coordination and balance.

International Cooperative Ataxia Rating Scale (ICARS) was developed by the Committee of the World Federation of Neurology in 1997 [15]. The scale has been validated for patients with degenerative cerebellar diseases and focal cerebellar lesions [13]. The score has excellent validity (internal consistency), high criterion-related reliability, and good test re-test reliability [12–14]. The semi-quantitative scale is composed of a total of 19 items and has four subscales: posture and gait disturbance, kinetic functions, speech, and oculomotor disorders. The ICARS structure is based on the functional–anatomical compartmentalization of the cerebellum. The higher the score (max. 100 points), the more severe is the ataxia/impairment [15]. The subscales differ in numbers of items and maximum scores for impairment.

The Scale for Assessment and Rating of Ataxia (SARA) was developed by a group of European neurologists in 2006 to assess the range of impairments in cerebellar ataxia with fewer items than the ICARS [13]. The score has been developed and validated in patients with spinocerebellar ataxia, Friedreich’s ataxia, chronic focal cerebellar lesions, and other ataxia disorders [13, 25]. The interrater reliability, test re-test reliability, and internal consistency were high [13, 25]. The *SARA* has eight items: gait, stance, sitting, speech disturbance, finger-chase-test, nose-finger-test, fast alternating hand movement, and heel-shin slide. The higher the score (max. 40), the higher the severity of ataxia [13, 26].

The German version of the Life Satisfaction Questionnaire (Fragebogen zur Lebenszufriedenheit, FLZ) were used to assess adult participants’ health and satisfaction status in different aspects of life [24]. Questionnaires were used to evaluate participants’ and parental education.

### Statistical Analyses

Data were analyzed using SPSS 26.0 (IBM SPSS Statistics for Windows, IBM Corp., Armonk, New York, USA). Differences between VP and term control children and adults in group characteristics were tested using *Χ*^2^ tests for categorical variables and independent-samples *t* tests or Mann–Whitney *U* tests for continuous variables. To assess whether VP children and adults had total scores above 0 more often, frequencies between VP and term born children and adults were compared using *Χ*^2^ tests, and odds ratios with 95% confidence intervals were computed. Group differences in ICARS and SARA total and subscale scores between VP and term control children and adults were tested using non-parametric Mann–Whitney *U* tests, given that most scores were not normally distributed. Effect sizes are reported as Cohen’s *r*: 0.10 = small, 0.30 = medium, 0.50 = large effects. In addition, Kendall’s *τ*_*b*_ correlations were calculated to determine associations between ICARS and SARA total scores among VP and term born children and adults, respectively, as well as associations between gestational age (in wks) and ARS scores for children and adults. The alpha level (initially set at *p* < 0.05) was adjusted for multiple testing using Bonferroni-Holm correction to avoid inflation of type 1 error and was two-tailed for all analyses.

### MRI Analysis

Neonatal MRI at term-equivalent age was performed in VP children as part of the clinical routine and were available for assessment. The total abnormality score (TAS) and the transcerebellar diameter were reviewed (TCD) in the neonatal MRI as MRI metrics predictive of neurodevelopmental outcome of very preterm-born children [27, 28]. In young adult brains, MRI scans were acquired on a 3 T Vida Siemens scanner as part of another study. Individual T1-weighted structural MRI volumes were segmented and labelled to Hammers adult brain atlas using the computational anatomy toolbox for SPM12 (CAT12, release 1742) on a Linux platform running MATLAB 9.6 [29]. Process yielded the total inner cranial volume. Cerebellar and brainstem sub-volumes were calculated by masking segmented maps with the respective atlas labels.

## Results

### ICARS and SARA Scores in Very Preterm and Term Born Children

**Fig. 1:**
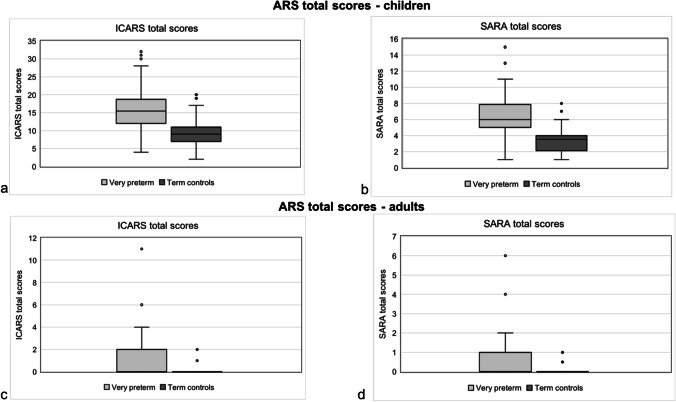
ICARS and SARA total scores in children and adults. **a**, **b** Boxplots of ICARS and SARA total scores of very preterm-born children compared to term-born controls. **c**, **d** ICARS and SARA total scores of very preterm-born young adults compared to term-born controls. Lower and upper box boundaries represent the 25th and 75th percentiles, respectively. The line inside the box displays the median. The lower and upper error lines mark the 10th and 90th percentiles, respectively. The filled circles represent data falling outside the 10th and 90th percentiles. ARS, ataxia rating scale

All children reached a score of 2 or higher in ICARS and 1 or higher in SARA. Mann–Whitney *U* tests showed that VP children had significantly higher ICARS and SARA total scores compared with their term born controls with large effect sizes (see Table 3, Fig. 1a, b). VP children scored significantly higher on all ICARS subscales, especially on *posture and gait disturbance* and *kinetic functions* with medium to large effect sizes. Comparisons survived the correction for multiple testing, except for the subscale *speech*. In addition, VP children had significantly higher scores in all SARA subscales compared with term born children, except for the subscale *sitting*. Group differences were most prominent in subscales *gait*, *fast alternating hand movement*, and *heel-shin slide*, representing medium-sized effects. Subscales *stances*, *speech disturbance*, and *nose-finger-test* did not survive the correction for multiple testing.

**Table 3:** Comparisons of ICARS and SARA total and subscale scores between very preterm and term born control children (*N* = 120)

		Term controls (*n* = 60)	Very preterm (*n* = 60)	*U*	*Z*	*p*^a^	*r*^c^
ICARS total score	*M* (*SD*)	9.17 (3.88)	15.98 (6.29)	582.50	− 6.40	< .001	− 0.584
	*Range*	2.00–20.00	4.00–32.00				
	*Mdn* (*IQR*)	9.00 (4.00)	15.50 (7.00)				
Posture and gait disturbance	*M* (*SD*)	0.30 (0.56)	1.47 (1.50)	771.00	− 5.86	< .001	− 0.535
	*Range*	0.00–2.00	0.00–8.00				
	*Mdn* (*IQR*)	0.00 (1.00)	1.00 (2.00)				
Kinetic functions	*M* (*SD*)	8.48 (3.88)	13.15 (4.97)	810.00	− 5.21	< .001	− 0.476
	*Range*	0.00–19.00	4.00–28.00				
	*Mdn* (*IQR*)	8.50 (5.00)	13.00 (6.00)				
Speech	*M* (*SD*)	0.00 (0.00)	0.17 (0.62)	1650.00	− 2.27	.023^b^	− 0.207
	*Range*	0.00–0.00	0.00–3.00				
	*Mdn* (*IQR*)	0.00 (0.00)	0.00 (0.00)				
Oculomotor disorders	*M* (*SD*)	0.38 (0.74)	1.22 (1.42)	1183.50	− 3.67	< .001	− 0.335
	*Range*	0.00–3.00	0.00–5.00				
	*Mdn* (*IQR*)	0.00 (1.00)	1.00 (2.00)				
SARA total scores	*M* (*SD*)	3.51 (1.54)	6.50 (2.53)	478.50	− 6.98	< .001	− 0.637
	*Range*	1.00–8.00	1.00–15.00				
	*Mdn* (*IQR*)	3.50 (1.90)	6.00 (2.90)				
Gait	*M* (*SD*)	0.17 (0.42)	0.72 (0.67)	934.50	− 5.32	< .001	− 0.486
	*Range*	0.00–2.00	0.00–3.00				
	*Mdn* (*IQR*)	0.00 (0.00)	1.00 (1.00)				
Stance	*M* (*SD*)	0.10 (0.30)	0.27 (0.45)	1500.00	− 2.35	.019 ^b^	− 0.215
	*Range*	0.00–1.00	0.00–1.00				
	*Mdn* (*IQR*)	0.00 (0.00)	0.00 (1.00)				
Sitting	*M* (*SD*)	0.00 (0.00)	0.00 (0.00)	1800.00	0.00	> .999	0.000
	*Range*	0.00–0.00	0.00–0.00				
	*Mdn* (*IQR*)	0.00 (0.00)	0.00 (0.00)				
Speech disturbance	*M* (*SD*)	0.00 (0.00)	0.13 (0.60)	1680.00	− 2.03	.043 ^b^	− 0.185
	*Range*	0.00–0.00	0.00–4.00				
	*Mdn* (*IQR*)	0.00 (0.00)	0.00 (0.00)				
Finger-chase-test	*M* (*SD*)	0.61 (0.46)	0.85 (0.42)	1338.50	− 2.87	.004	− 0.262
	*Range*	0.00–1.00	0.00–2.00				
	*Mdn* (*IQR*)	1.00 (1.00)	1.00 (0.00)				
Nose-finger-test	*M* (*SD*)	0.23 (0.40)	0.46 (0.54)	1407.50	− 2.44	.015 ^b^	− 0.223
	*Range*	0.00–1.00	0.00–2.00				
	*Mdn* (*IQR*)	0.00 (0.50)	0.00 (1.00)				
Fast alternating hand movement	*M* (*SD*)	1.75 (0.91)	2.69 (0.79)	841.50	− 5.31	< .001	− 0.485
	*Range*	0.00–3.00	0.50–4.00				
	*Mdn* (*IQR*)	2.00 (1.80)	3.00 (1.00)				
Heel-shin slide	*M* (*SD*)	0.66 (0.74)	1.25 (1.01)	1174.00	− 3.47	.001	− 0.317
	*Range*	0.00–3.00	0.00–4.00				
	*Mdn* (*IQR*)	0.50 (1.00)	1.00 (1.00)				

Data are presented as mean (standard deviation), range, and median (interquartile range). *M*, mean; *SD*, standard deviation; *Mdn*, median; *IQR*, interquartile range

^a^Two-tailed significance based on non-parametric Mann–Whitney *U* tests

^b^Group difference not significant after adjusting for multiple testing using Bonferroni-Holm correction

^c^Effect size estimate *r*

**Fig. 2:**
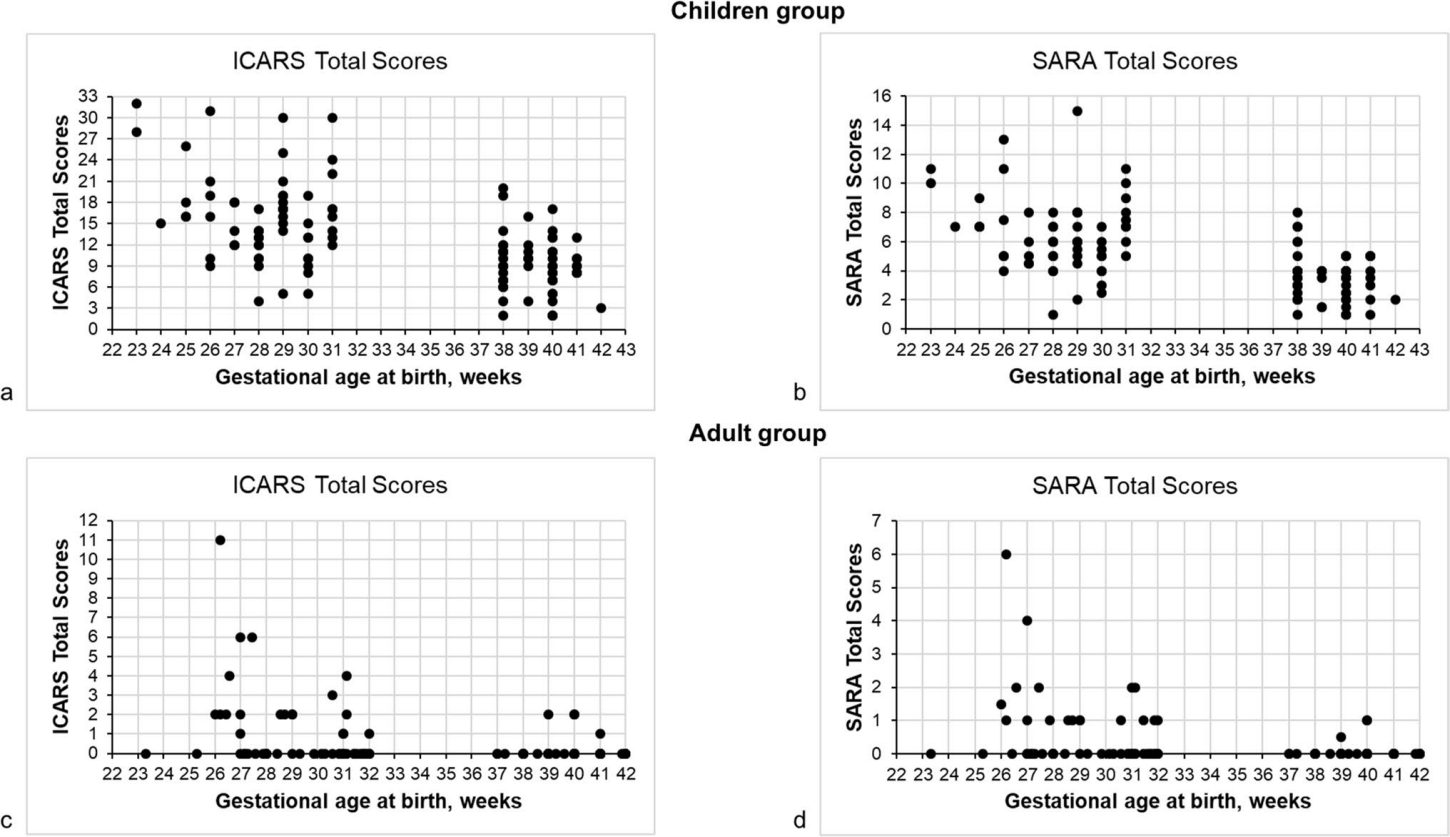
Association between the distribution of (**a**) the ICARS and (**b**) the SARA total scores and gestational age at birth in weeks for children and between the distribution of (**c**) the ICARS and (**d**) the SARA total scores and gestational age at birth in weeks for adults

There was a strong, negative correlation between gestational age in weeks at birth and ICARS (*τ*_*b*_ =  − 0.413, 95% CI [− 0.508, − 0.308], *p* < 0.001) and SARA total scores (*τ*_*b*_ =  − 0.444, 95% CI [− 0.535, − 0.342], *p* < 0.001) (Fig. 2). The ICARS total scores were strongly correlated with the SARA total scores in VP children and controls (VP children: *τ*_*b*_ = 0.645, 95% CI [0.533, 0.735], *p* < 0.001; term controls: *τ*_*b*_ = 0.440, 95% CI [0.290, 0.568], *p* < 0.001).

### ICARS and SARA Scores in Very Preterm and Term Born Adults

VP adults invariably scored above 0 compared to controls based on ICARS total scores (33.9% vs. 6.3%, *Χ*^2^(1) = 14.77, *p* < 0.001, OR = 7.70, 95% CI [2.43–24.41]) and SARA total scores (32.1% vs. 4.7%, *Χ*^2^(1) = 15.59, *p* < 0.001, OR = 9.63, 95% CI [2.66–34.91]).

VP adults had significantly higher ICARS and SARA total scores compared to term born controls (see Table 4, Fig. 1c, d) with medium-sized effects. VP adults scored significantly higher on ICARS subscale *posture and gait disturbance* with a small effect size. There were no significant differences on other ICARS subscales between VP and term born control adults, after adjusting analyses for multiple testing. In addition, VP adults had significantly higher scores in SARA subscales *stance*, *heel-shin slide*, and *speech disturbance*, indicating small-sized effects, whereas there were no significant differences on other SARA subscales between the groups. However, none of the group differences in SARA subscales survived the correction for multiple testing.

**Table 4:** Comparisons of ICARS and SARA total and subscale scores between very preterm and term born control adults (*N* = 120)

		Term controls (*n* = 64)	Very preterm (*n* = 56)	*U*	*Z*	*p*^a^	*r*^c^
ICARS total score	*M* (*SD*)	0.11 (0.44)	1.00 (1.99)	1285.50	− 3.88	< .001	− 0.354
	*Range*	0.00–2.00	0.00–11.00				
	*Mdn* (*IQR*)	0.00 (0.00)	0.00 (2.00)				
Posture and gait disturbance	*M* (*SD*)	0.02 (0.13)	0.25 (0.58)	1498.00	− 3.09	.002	− 0.282
	*Range*	0.00–1.00	0.00–2.00				
	*Mdn* (*IQR*)	0.00 (0.00)	0.00 (0.00)				
Kinetic functions	*M* (*SD*)	0.09 (0.43)	0.57 (1.37)	1489.00	− 2.78	.005^b^	− 0.254
	*Range*	0.00–2.00	0.00–8.00				
	*Mdn* (*IQR*)	0.00 (0.00)	0.00 (0.00)				
Speech	*M* (*SD*)	0.00 (0.00)	0.18 (0.58)	1632.00	− 2.43	.015^b^	− 0.222
	*Range*	0.00–0.00	0.00–2.00				
	*Mdn* (*IQR*)	0.00 (0.00)	0.00 (0.00)				
Oculomotor disorders	*M* (*SD*)	0.00 (0.00)	0.00 (0.00)	1792.00	0.00	> .999	0.000
	*Range*	0.00–0.00	0.00–0.00				
	*Mdn* (*IQR*)	0.00 (0.00)	0.00 (0.00)				
SARA total scores	*M* (*SD*)	0.04 (0.18)	0.54 (1.08)	1284.00	− 4.04	< .001	− 0.369
	*Range*	0.00–1.00	0.00–6.00				
	*Mdn* (*IQR*)	0.00 (0.00)	0.00 (1.00)				
Gait	*M* (*SD*)	0.00 (0.00)	0.05 (0.23)	1696.00	− 1.87	.062	− 0.171
	*Range*	0.00–0.00	0.00–1.00				
	*Mdn* (*IQR*)	0.00 (0.00)	0.00 (0.00)				
Stance	*M* (*SD*)	0.00 (0.00)	0.11 (0.31)	1600.00	− 2.68	.007^b^	− 0.245
	*Range*	0.00–0.00	0.00–1.00				
	*Mdn* (*IQR*)	0.00 (0.00)	0.00 (0.00)				
Sitting	*M* (*SD*)	0.00 (0.00)	0.00 (0.00)	1792.00	0.00	> .999	0.000
	*Range*	0.00–0.00	0.00–0.00				
	*Mdn* (*IQR*)	0.00 (0.00)	0.00 (0.00)				
Speech disturbance	*M* (*SD*)	0.00 (0.00)	0.11 (0.37)	1632.00	− 2.43	.015^b^	− 0.222
	*Range*	0.00–0.00	0.00–2.00				
	*Mdn* (*IQR*)	0.00 (0.00)	0.00 (0.00)				
Finger-chase-test	*M* (*SD*)	0.01 (0.06)	0.04 (0.16)	1723.50	− 1.16	.247	− 0.106
	*Range*	0.00–0.50	0.00–1.00				
	*Mdn* (*IQR*)	0.00 (0.00)	0.00 (0.00)				
Nose-finger-test	*M* (*SD*)	0.03 (0.15)	0.12 (0.32)	1646.50	− 1.60	.110	− 0.146
	*Range*	0.00–1.00	0.00–1.00				
	*Mdn* (*IQR*)	0.00 (0.00)	0.00 (0.00)				
Fast alternating hand movement	*M* (*SD*)	0.00 (0.00)	0.02 (0.13)	1760.00	− 1.07	.285	− 0.098
	*Range*	0.00–0.00	0.00–1.00				
	*Mdn* (*IQR*)	0.00 (0.00)	0.00 (0.00)				
Heel-shin slide	*M* (*SD*)	0.00 (0.00)	0.11 (0.31)	1600.00	− 2.68	.007^b^	− 0.245
	*Range*	0.00–0.00	0.00–1.00				
	*Mdn* (*IQR*)	0.00 (0.00)	0.00 (0.00)				

Data are presented as mean (standard deviation), range, and median (interquartile range). *M*, mean; *SD*, standard deviation; *Mdn*, median; *IQR*, interquartile range

^a^Two-tailed significance based on non-parametric Mann–Whitney* U* tests

^b^Group difference not significant after adjusting for multiple testing using Bonferroni-Holm correction

^c^Effect size estimate *r*

There was an average, negative correlation between gestational age in weeks at birth and ICARS (*τ*_*b*_ =  − 0.318, 95% CI [− 0.422, − 0.206], *p* < 0.001) and SARA total scores (*τ*_*b*_ =  − 0.317, 95% CI [− 0.421, − 0.206], *p* < 0.001) (Fig. 2).

The ICARS total scores were strongly correlated with the SARA total scores among VP adults and controls (VP adults: *τ*_*b*_ = 0.745, 95% CI [0.654, 0.815], *p* < 0.001; term controls: *τ*_*b*_ = 0.863, 95% CI [0.814, 0.900], *p* < 0.001).

### MRI Data

The mean TAS in VP children was low (2.9) according to Dewan et al. (2019). Transcerebellar diameter (TCD) in VP children was 50.9 mm which is considered to be normal according to Kidokoro et al. (2013) (see Table 1) [30].

In the adult group, the cerebellar volumes did not differ in the young adult group between VP and controls (see Table 2).

Neither the children nor the young adult group showed abnormalities or pathologies in the MRI.

## Discussion

Very preterm (VP) children and adults had higher total scores in ataxia rating scales (ARS) compared to term controls. Preterm birth is associated with higher scores in ARS. In addition, differences prevailed into young adulthood. Both scales were equally able to identify group differences with effects sizes being larger in children. Compared to controls, scores in the subscale *posture and gait* showed most consistent differences between groups. Gestational age at birth was negatively associated with ARS total scores in both children and adults.

In line with our findings, previous studies found a 3–4 times increased risk of mild to moderate motor problems in VP children compared to the general pediatric population [5]. VP adults had an 8–9 times increased likelihood to score above 0 in ARS compared to controls, which suggests an increased risk of difficulties in coordination and balance.

ARS offer easy to apply, standardized, and quantifiable instruments to identify soft neurological signs of difficulties in coordination and balance.

Of note, healthy children also show higher scores in ARS up to an age of 12 years [16, 17], which are likely due to the development of the sensory-motor systems [9, 10]. In line with this, healthy term-born children also scored higher in ARS in this study [16, 17]. However, both VP children and adults scored significantly higher in the ARS than controls, indicating deficits in coordination and balance, which may be explained by an aberrant development of brain areas involved in motor control including but not limited to the cerebellum. Dewey et al. described that preterm children at risk for DCD had volume reduction in motor areas including cerebral cortex, cerebellum, and basal ganglia [19]. Furthermore, Lahti et al. described an association between diffusion tensor matrix at term-equivalent age of the corpus callosum, corona radiata, and optic radiation with motor performance in 11-year-old preterm children [31]. Alterations in these brain regions are well known in VP infants, referred as encephalopathy of prematurity [3]. In addition to cerebral injury, there is increasing evidence of cerebellar involvement [3]. The most common alteration associated with prematurity is a symmetric volume reduction of the cerebellar hemispheres without focal lesions [32, 33]. Tam et al. demonstrated an association between volume reduction on magnetic resonance imaging (MRI) at term-equivalent age and an 8–10 times fold increased risk for truncal hypotonia, postural instability, and patellar hyperreflexia at the age of 18 months [33].

VP adults scored significantly higher in ARS than controls indicating that difficulties in coordination and balance may prevail until young adulthood. The medial region of the cerebellum (vermis) plays a central role in balance and locomotion [34, 35]. The vermis of the cerebellum shows a selective vulnerability for hypoxia especially in neonates [36]. This may explain why impairments in posture and gait prevail in adulthood in VP born individuals. Likewise, impairments of posture and gait are the most common symptoms in degenerative cerebellar ataxias [37].

Persisting motor problems were also reported in a Norwegian cohort study of very low birth weight born children. Evensen et al. discovered that in the preterm group, one out of four children had motor problems in the M-ABC (scores below the 5th percentile), persisting until 23 years of age [38].

Mild motor problems, unexplained by neurological conditions, with an impact on daily activities and age-appropriate participation are described as DCD [21]. Difficulties in coordination and balance are among the symptoms of DCD, for which VP children are especially at risk [18, 21]. However, there is a lack of clearly defined diagnostic criteria and diagnostic tools to confirm this diagnosis. Children with DCD and their families often have long time- and cost-consuming ways until the diagnosis is defined.

The application of ARS reliably revealed difficulties in coordination and balance in VP participants in the present study. Thus, it may a useful screening tool to decide which children should be tested with more extended instruments to diagnose motor problems such as the M-ABC-2 and BOT-2 [23].

We could also show a high correlation between total scores of both ARS. Whereas ICARS takes approximately 10–15 min to apply, SARA is briefly assessed in 4–5 min, but does not include oculomotor function [13, 15]. Since SARA takes only one-third of the time required for ICARS, it may be most suitable to be integrated into follow-up care to screen for cerebellar dysfunction with difficulties in coordination and balance.

Children’s motor abilities and physical performance have been found to affect their self-esteem, mental health, academic achievement, and acceptance by peers [19, 22, 39]. VP children have a 3–4 times fold increased risk of developing psychiatric disorders [6]. Thus, being clumsy and having significant deficits in coordination and balance, may put preterm children at further risk of social-emotional maladjustment, including difficulties with peers [40]. It is of great importance to identify children with these problems to intervene early and prevent later maladjustment [18, 20, 21, 39, 40]. Increased awareness and knowledge of health-care professionals involved in the follow-up care of VP children may help to apply early interventions, which improve coordination and balance in physical training. Interventions may not only train VP children’s physical abilities but, in turn, may also increase their self-esteem and social-emotional development.

The aftermath of perinatal impairment of cerebellar growth/cerebellar injury came into focus during the last decade. In addition to motor impairment, the developmental dysfunction of the cerebellum seems to have a great impact on long-term cognitive, behavioral, and social-emotional development of individuals [41]. Prematurity appears to be associated with a developmental form of the cerebellar cognitive affective syndrome [41]. Therefore, it would be reasonable to assess VP born adults with the Cerebellar Cognitive Affective Syndrome Scale (CCAS-S) [42], and to develop a CCAS-S for children.

This study has some limitations: First we did not follow one sample to investigate ARS longitudinally from childhood into adulthood. However, we increased comparability between children and adult groups by recruiting participants based on the same inclusion and exclusion criteria. Second, high scores in ARS are not exclusively indicative of cerebellar dysfunction but may also indicate other brain pathologies, such as injuries in basal ganglia or motor cortex, and symptoms of impaired proprioception, muscle tone, vestibular- and visual-motor control [43]. Third, a dose–response relation between ARS scores and gestational age was not done across the whole gestation spectrum. Further investigations with larger sample sizes and longitudinal studies of VP children are needed and planned.

## Conclusions

VP children showed significantly higher scores in ARS than controls. These differences were also present in VP adults, suggesting that deficits likely prevail until adulthood. ARS are a time- and cost-effective method to unmask these differences and to screen for difficulties in coordination and balance in a patient group at risk. Since SARA takes only one-third of the time required for ICARS, it may be most suitable to be integrated into follow-up care. Data suggest that ARS may be useful screening tools to decide which patients should be tested with more extended motor scales such as the M-ABC-2.
